# A Study to Investigate the Role of Noncoding RNA miR146 Alpha as a Potential Biomarker in Prostate Cancer

**DOI:** 10.30683/1927-7229.2022.11.03

**Published:** 2022-07-22

**Authors:** Myla Worthington, Chelsey Aurelus, Narendra Banerjee, Christopher Krauss, William Kahan, Satyendra Banerjee, Sherita Gavin, Victoria Bartlett, Gloria Payne, Jeffrey Rousch, Mukesh Verma, Fazlul Sarkar, Hirendra Nath Banerjee

**Affiliations:** 1 Department of Natural Sciences, Elizabeth City State University, University of North Carolina, NC 27909, USA; 2 Division of Cancer Control and Population Sciences, National Cancer Institute, National Institutes of Health, Suite 4E102, 9609 Medical Center Drive, Bethesda, USA; 3 Department of Pathology, Wayne State University and Barbara Karmanos Cancer Center, Detroit, USA

**Keywords:** Prostate Cancer, Non Coding RNA, miR146 alpha, Biomarker

## Abstract

There is a need for additional biomarkers for the diagnosis and prognosis of prostate cancer. MicroRNAs are a class of non-protein coding RNA molecules that are frequently dysregulated in different cancers including prostate cancer and show promise as diagnostic biomarkers and targets for therapy. Here we describe the role of micro RNA 146 a (miR-146a) which may serve as a diagnostic marker for prostate cancer, as indicated from the data presented in this report. Also, a pilot study indicated differential expression of miR-146a in prostate cancer cell lines and tissues from different racial groups. This report provides a novel insight into understanding the prostate carcinogenesis.

## INTRODUCTION

Although biomarkers exist for prostate cancer diagnosis, either they have poor sensitivity and specificity, or they cannot detect prostate cancer early enough to make strategies for proper treatments. Only prostate specific antigen (PSA) is a biomarker which is used extensively in the clinic and it can be only used in those cases where the levels of PSA are more than 4 ng/ml. Different forms of PSA (free PSA, total PSA, PSA velocity) have been characterized but the advantage of one over the others is not convincing [1].

Cancer is a genetic and epigenetic disease and epigenetic regulation has been observed in all major tumor types studied to date including prostate cancer [2–4]. The four major components of epigenetics are DNA methylation, histone modifications, non-coding RNA (mostly microRNAs) expression and chromatin modulation [1, 5–9]. In the last few years, tremendous progress has been made in understanding the biogenesis of miRNAs and their role in altering the translation of messenger RNA by binding to them or degrading them. Compared to mRNAs, microRNAs are shorter in length and have a longer half-life (due to their secondary structure and size) which makes them stable and potentially reliable as clinical diagnostic and prognostic biomarkers [10, 11]. Whether the miRNAs act as tumor suppressors or oncogenes, dysregulated miRNAs allow cells to escape from regulatory control and contribute to tumor formation [12, 13]. Enabling early detection and aggressive treatment of rapidly recurrent and refractory phenotypes would reduce disease mortality. Dysregulated miRNAs are also potentially viable novel targets of prostate cancer. miRNAs either come in the circulation as free-floating bodies or they are delivered via exosomes (small molecules with bipolar membrane [14, 15].

Noncoding RNA miR146 alpha has been reported to be upregulated in many types of cancer [11], we report our findings on miR146 alpha expression in various prostate cancer cell lines in comparison to normal prostate cells and from matched control, prostate cancer biopsy samples from a cohort of patients at Wayne State University Cancer Center, Detroit, USA.

## MATERIALS AND METHODS

### Cell Culture and Maintenance

Cell culture and maintenance were described in our previous publication [27] PC3, LNCAP, RWPE1, and EO6 AA cell lines were obtained from the American Type Culture Collection. The cell lines were grown and maintained in Roswell Park Memorial Institute Medium (RPMI 1640). Cells were stored at 5% CO2 in 25cm2 filter cap flasks in a laboratory CO2 incubator and were additionally maintained with 10% Fetal Bovine Serum (FBS) and 100μl/ml antibiotic/antimycotic (penicillin/streptomycin/ amphotericin B Type Culture Collection (Manassas, VA). RWPE1 are normal prostate cell line, c42b is a white prostate cancer line and EO6AA is African American prostate cancer cell line.

### Patients and Prostate Tissue Specimen Collection

After obtaining institutional review board approval, retrospective archival pre-treatment PCa tissues and matched adjacent normal tissues were obtained from the Biospecimen Core of Karmanos Cancer Institute (KCI) collected from patients who underwent radical prostatectomy at KCI. We also obtained PCa tissue specimens from Henry Ford Health System (HFHS), Detroit, Michigan. Patients’ clinical characteristics were obtained from the hospital database including race. Pathological features were ascertained from microscopic evaluation of tumor slides by pathologists both at KCI and at HFHS. Gleason score (grade) was obtained in each case from the clinical database.

### Real-Time RT-PCR

For testing the miRNA levels, the total RNA from cells and tissues was isolated using the miRNeasy Mini Kit (Qiagen) and the DNA was removed using an RNase-free DNAase kit (Qiagen). 20 ng of RNA were reverse transcribed into cDNA using a Universal cDNA Synthesis Kit (Exiqon, Woburn, MA) according to the manufacturer’s instruction. Real time PCR was performed using specific miRNA primers (Exiqon) to quantify miRNA expression by using SYBR Green RTPCR Reagents (Applied biosystems). The relative amount of miRNA was normalized to the expression of RNU1a1.

## RESULTS

**Figure 1:**
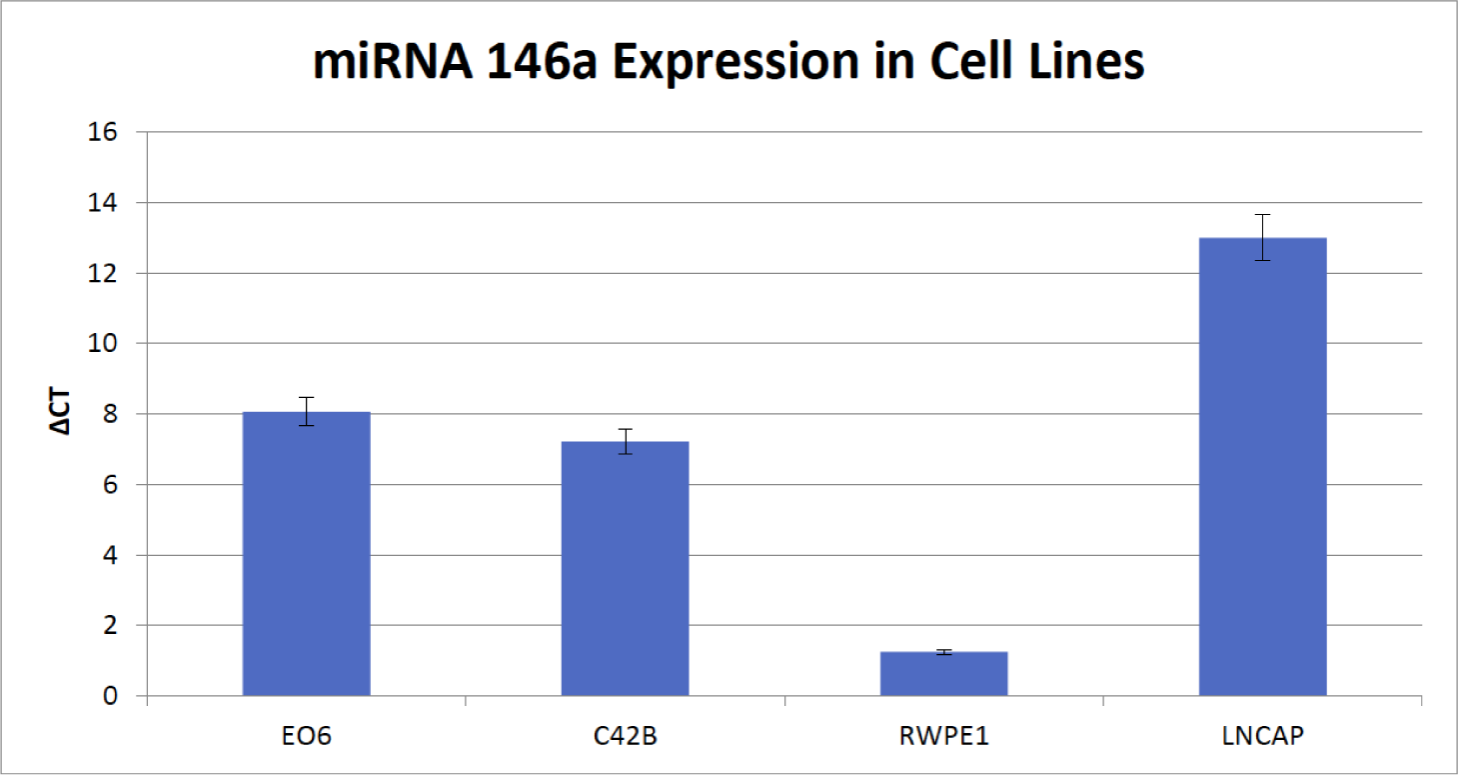
The Real Time PCR experiment measuring the expression of MiRNA 146a in multiple cell lines shows that MiRNA 146a in expressed less in normal cell line RWPE1 than all the other cancer cell lines. The Single Factor Anova reveals that the results are statistically significantly different. (Anova: Single Factor, a= 0.05).

**Figure 2:**
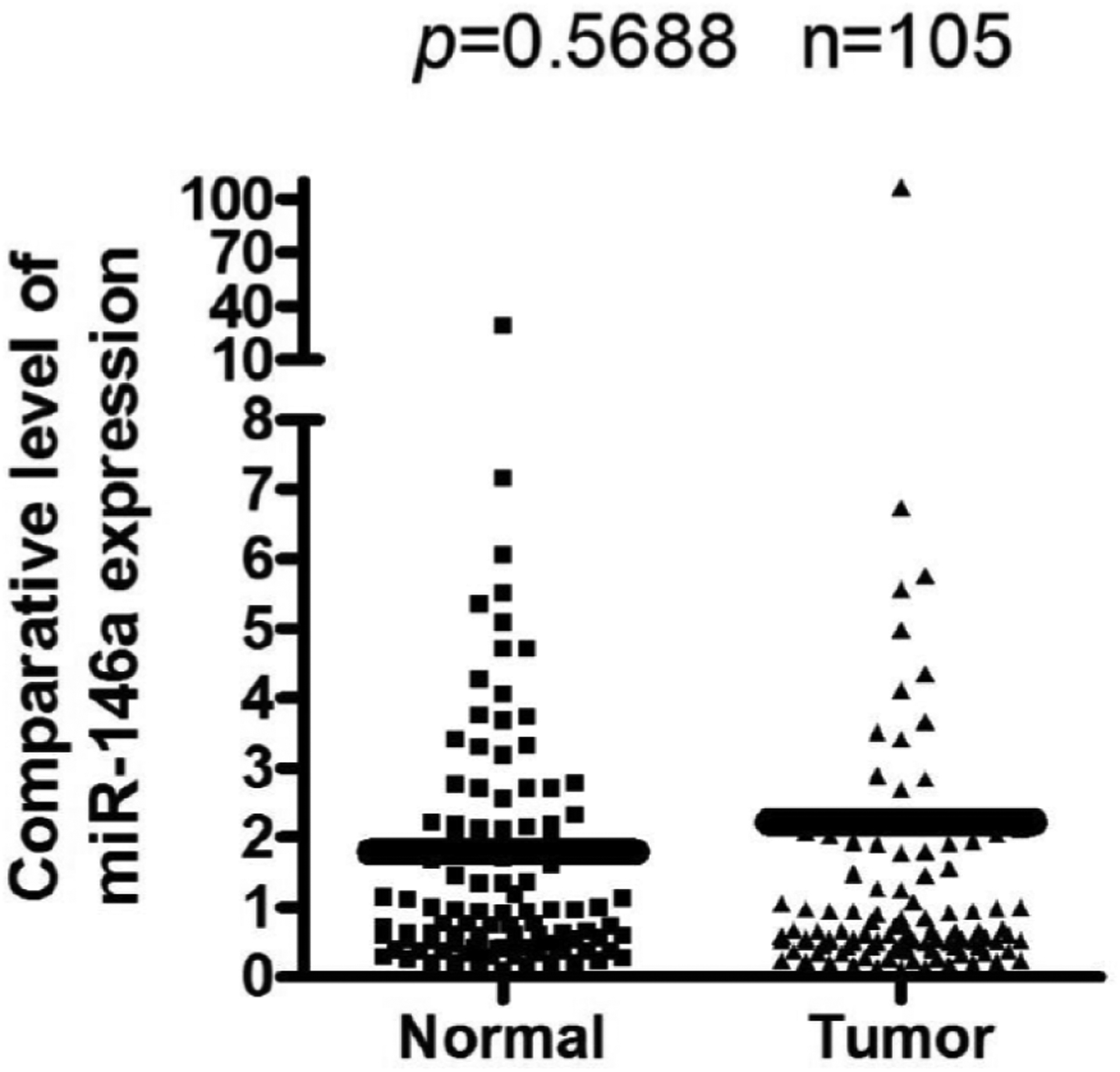
The expression level of miR-146a was higher in prostate cancer tissues compared to normal tissues. Total RNAs were extracted from prostate cancer tissues and adjacent normal prostate tissues. The patients are from all races and with all grades of prostate cancer. The expression of miR-146a in tumor and normal tissues was accessed by real-time RT-PCR assay (Exiqon). The expression level of miR-146a was normalized with the level of RNU1a1.

Levels of miR-146a was found to increase in all prostate cancer cell lines compared to normal cell line RWPE1 (Figure 1). Investigating whether similar results were seen in patient samples, expression of miR-146a was analyzed in prostate cancer tissues and compared with normal matched control tissue biospecimens, Increased expression of miR-146a was observed in prostate tumor tissues compared to those from matched control biopsy samples (Figure 2).

## DISCUSSION AND CONCLUSION

The role of miR-146a-5p as an oncomiR was first identified in a study by Ye *et al*., wherein the overexpression of miR-146a-5p was associated with the aggressiveness of PTC [16]. In lung cancer, Wortchosfky *et al*. showed that the high expression level of miR-146b was predictive of recurrence [17]. Li *et al*. found that miR-141–5p was an oncogenic miRNA in prostate cancer [18]. However, there are conflicting reports that support the role of miR-146a-5p as a tumor suppressor. miR-146a-5p was thought to function as a suppressor miRNA and its expression correlated with the prognosis of non-small cell lung cancer. These contrasting roles of miR-146a-5p may be associated with the different targets repressed by this miRNA in different tissues as well as the regulation of its functions via a complex network in a tissue- and stage dependent manner. The precise molecular mechanisms underlying miR-146a-5p-mediated regulation of cancer are unknown. In this study, we found both in prostate cancer cell lines and biopsy samples, upregulation of miR-146a and its oncoMir potential, more investigations need to be required to find the mechanisms and core canonical pathways involved in this oncogenic role of miR-146a and its diagnostic and prognostic values in prostate cancer.
